# Differential expression of NPM, GSTA3, and GNMT in mouse liver following long-term *in vivo* irradiation by means of uranium tailings

**DOI:** 10.1042/BSR20180536

**Published:** 2018-10-17

**Authors:** Lan Yi, Hongxiang Mu, Nan Hu, Jing Sun, Jie Yin, Keren Dai, Dingxin Long, Dexin Ding

**Affiliations:** 1 Key Discipline Laboratory for National Defense for Biotechnology in Uranium Mining and Hydrometallurgy, University of South China, Hengyang 421001, Hunan Province, P.R. China; 2 Institute of Cytology and Genetics, College of Pharmaceutical and Biological Science, University of South China, Hengyang 421001, Hunan Province, P.R. China

**Keywords:** chronic gamma radiation, C57BL/6J mice, liver, protein expression, uranium tailings

## Abstract

Uranium tailings (UT) are formed as a byproduct of uranium mining and are of potential risk to living organisms. In the present study, we sought to identify potential biomarkers associated with chronic exposure to low dose rate γ radiation originating from UT. We exposed C57BL/6J mice to 30, 100, or 250 μGy/h of gamma radiation originating from UT samples. Nine animals were included in each treatment group. We observed that the liver central vein was significantly enlarged in mice exposed to dose rates of 100 and 250 μGy/h, when compared with nonirradiated controls. Using proteomic techniques, we identified 18 proteins that were differentially expressed (by a factor of at least 2.5-fold) in exposed animals, when compared with controls. We chose glycine N-methyltransferase (GNMT), glutathione S-transferase A3 (GSTA3), and nucleophosmin (NPM) for further investigations. Our data showed that GNMT (at 100 and 250 μGy/h) and NPM (at 250 μGy/h) were up-regulated, and GSTA3 was down-regulated in all of the irradiated groups, indicating that their expression is modulated by chronic gamma radiation exposure. GNMT, GSTA3, and NPM may therefore prove useful as biomarkers of gamma radiation exposure associated with UT. The mechanisms underlying those changes need to be further studied.

## Introduction

The biological effects of chronic low dose rate on normal tissues have attracted much attention in recent years [1]. Many studies have shown that sustained low dose radiation (LDR) can cause harm to organisms, such as chromosome aberrations [2], genomic instability [3], cell inactivation [4], and tumorigenicity [5]. Persistent detrimental effects for the cardiovascular system have also been reported [6]. There is also evidence to suggest that ionizing radiation, in addition to other factors, may have an impact on the pathology of neurodegenerative diseases such as Alzheimer’s [7]. However, there is also a large body of literature describing the beneficial effects of LDR exposure, such as the prevention of doxorubicin-induced cardiotoxicity by suppressing mitochondrial-dependent oxidative stress and apoptosis signaling [8]. Research has shown that LDR can activate the adaptive immune response, promoting immune-dependent tumor inhibition [9].

Recently, there has been a growing interest in environmental and public health impact of uranium tailings (UT), with uranium being shown as the cause of irreversible impacts on the surrounding environment [10]. UT can cause environmental pollution [11,12] and represent a hazard for humans [13,14]. Research has shown that chemicals and radionuclides present in the tailings influence soil properties and microbial diversity [15]. UT can lead to human disease and health complications after occupational or environmental exposure [16]. Thus, it is very important to develop biomarkers that can be used to assess risks associated with UT.

Proteomics provides information on the structure, expression, and function of proteins. It has been applied in fields such as clinical diagnosis [17–19] and tumor marker discovery [20]. Proteomics can be used to analyze the post-translational modifications of proteins. It is used to identify and quantify the overall protein content of a cell, tissue or an organism, and it is one of the most informative methodologies available for comprehending gene function [21].

Through proteomics analysis, our previous research identified differentially expressed proteins in the liver tissues of mice that had received different doses of Cs-137 radiation for 90 and 180 days [22,23]. It is well known that α and γ radiations associated with UT also have the potential to harm workers. In the present study, we used UT as a low dose rate source to irradiate C57BL/6J mice. The purpose of the study was to use quantitative proteomics to identify liver biomarkers of radiation exposure for the early detection of UT-associated health effects. At first, we used quantitative proteomics to identify those proteins in mouse liver tissue that are differentially expressed. Then, we further analyzed selected proteins to evaluate if they could potentially be used as biomarkers of radiation exposure.

## Materials, design, and methods

### Mice and irradiation

We purchased 6-to 7-week-old male C57BL/6J mice from the Hunan Slack King of Laboratory Animal Co., Ltd. (Changsha, China). We bred the mice in the Animal Department of the University of South China. To initiate the study, we used 36 six- to seven-week-old mice that were randomly distributed between the four experimental groups. Three groups were exposed for 500 days to whole-body radiation from UT at dose rates of 30, 100, and 250 μGy/h. The UT dose rate was monitored precisely using a γ detector (Shanghai Renri Radiation Protection Equipment Co., Ltd., Shanghai, China). Nonirradiated mice, which were used as controls, were treated the same as the irradiated mice. The exposed mice were placed above the UT for 22 h per day. The remaining 2 h were used for clinical observations of the test animals, room cleaning and bedding replacement, and to provide a fresh supply of food and water. We used a lead brick to shield the nonirradiated mice. The dose rate received by the nonirradiated matched group was less than 0.2 μGy/h. Mice were housed at a temperature of 22–26°C, and humidity of 45–70%. After 500 days, mice were killed by cervical dislocation. The liver tissue was harvested, washed with PBS, and snap-frozen at −80°C for later use. We conducted all experimental procedures in accordance with the guidelines approved by the Institutional Animal Care and Use Committee of the University of South China, Hunan, China.

### Hematoxylin and eosin staining of liver tissues

The liver tissues were fixed in 10% neutral formalin solution, and embedded in paraffin, and then cut into 4-μm-thick sections. The sections transferred onto glass slides. The slides were immersed in H_2_O for 30 s and dipped in Mayer’s hematoxylin and agitated for 30 s. The slides were rinsed in H_2_O for 60 s and then stained with 1% eosin Y solution for 10–30 s with agitation. The sections were dehydrated with 95 and 100% alcohol for 30 s each, and then the alcohol was extracted with xylene. One or two drops of mounting medium was added and covered with a cover slip. All reagents came from Shanghai Biotechnology.

### Protein extraction and quantification

We homogenized whole frozen mouse livers to a powder using a liquid nitrogen-cooled pestle and mortar. The tissue powder was then rehydrated in lysis buffer and subjected to sonication. The resulting lysate was centrifuged at 1200 ***g*** for 20 min at 4°C, the supernatant was then collected and incubated on a shaking table at 4°C for 1 h. We used the Bicinchoninic Acid (BCA) Protein Assay Kit (Beijing ComWin Biotech Co., Ltd., Beijing, China) to quantify the proteins, and the supernatants were stored at −20°C.

### Two-dimensional gel electrophoresis, staining, and image analysis

We extracted the total protein from mouse liver of four groups for two-dimensional gel electrophoresis (2-DE) analysis [24]. After electrophoresis, Coomassie brilliant blue was used to stain gels, and we used PD Quest software (Bio-Rad, CA, U.S.A.) to perform background subtraction, spot detection, spot matching, and quantification. Each spot was analyzed with six gel images: three used the matched group and three used a group with a different dose of irradiation. We used a Powerlook1100, scanner, and UMAX (Taipei, China) to obtain gel images at 300 dots per inch (dpi), and then used GE Healthcare ImageMaster^™^ 2D Platinum 5.0 software (Stockholm, Sweden) to analyze the gel images. We used the 2D Elite Automatic Spot Detection Program to detect spots to calculate spot volumes with respect to normalization and background values. The percentage volume of gel occupied by each spot was determined by comparing spot volume with total gel volume. Student’s *t* test was used to determine the differentially expressed protein spots between control and experimental groups, spots with a fold-change of ≥2.5 and a *P*-value of <0.05 were considered statistically significant.

### *In situ* protein digestion and matrix-assisted laser desorption/ionization time of flight mass spectrometry

Prior to mass spectrometry analysis, protein spots were subjected to enzymatic hydrolysis followed by destaining and drying. Two standards were used to obtain the final peptide mass fingerprinting (PMF) and to calibrate the spectrum. The internal standard was the trypsin self-degradation ion peak and matrix peak. The external standard used was the Peptide Calibration Standard II from Bruker Daltonics, Inc. (Massachusetts, U.S.A.). Peak list generation was conducted using the GPS Explorer Software Version 3.6 (Applied Biosystems, Foster, U.S.A.), and searches against the international protein index (IPI) mouse protein databases were performed using the Mascot database search algorithms (version 2.1, Matrix Science Ltd). The identification of proteins by PMF using Mascot was carried out using parameters similar to those used for the IPI mouse database. We used the IPI mouse protein and Mascot database search engine to determine gene ontology categories, protein functions, and gene names. Proteins with scores >55 and located outside of the green shadow were considered to be meaningful ‘hits’ in the present study.

### Real-time polymerase chain reaction

Real-time PCR was performed to evaluate changes in glycine N-methyltransferase *(Gnmt)*, glutathione S-transferase A3 *(Gsta3)*, and nucleophosmin *(Npm1)* gene expression. *Actb* expression was used as an internal control. All primer sequences were designed using Primer 5.0 software and synthesized by Sangon Biotech (Shanghai, Japan). Primer sequences are shown in Table 1.

**Table 1:** The primer sequences for GNMT, GSTA3, NPM, and *Actb*

Gene	Primer sequence	Length
*Gnmt*	F: 5′AACTGGTTGACGCTGGACAA-3′	133 bp
	R: 5′TGTTCTTTAGTGCCAGCCGG-3′	
*Gsta3*	F: TCGACGGGATGAAACTGGTG	128 bp
	R: CAGATCCGCCACTCCTTCTG	
*Npm1*	F: GAAGTGTGGTTCAGGGCCTG	122 bp
	R: ATCGCTTTCCAGACATGCCT	
*Actb*	F: 5′ GCCAACCGTGAAAAGATGAC 3′	541 bp
	R: 5′ GAGGCATACAGGGACAGCAC 3′	

Total RNA was extracted from tissue samples using the High Pure RNA Tissue Kit (Omega Company, Norcross, U.S.A.) according to the manufacturer’s instructions. A NanoDrop ND-1000 (Omega Company) was used to measure RNA purity and concentration. The Transcriptor First Strand cDNA Synthesis Kit (Roche) was used to generate cDNA according to the manufacturer’s instructions. Briefly, total RNA was incubated with 1 µl oligo (dT)18 at 70°C for 3 min and cooled to 37°C for an additional 10 min. Subsequently, 4 µl dNTP mixture, 2 µl 10× RT reaction buffer (DBI Bioscience, Ludwigshafen, Germany), 200 units of Moloney murine leukemia virus reverse transcriptase, and 1 µl of RNase inhibitor were added and first strand cDNA synthesis then performed by incubating the mixture at 37°C for 1 h and then at 95°C for 5 min, followed by cooling on ice. Reverse-transcription and real-time PCR were performed as described previously [22]. Thermocycling parameters involved a 95°C incubation for 5 min and then 40 cycles of a 95°C incubation for 10 s, a 59°C incubation for 15 s and a 72°C incubation for 20 s. The samples were heated from 72–99°C to provide a melt curve analysis.

### Western blotting

The BCA protein quantification assay was used to measure liver total protein from irradiated and nonirradiated mice. Total protein (50 μg) was mixed with loading buffer at a 5:1 ratio. Samples were boiled for 5 min at 100°C to denature proteins, and then resolved by SDS–PAGE using a 10% polyacrylamide gel. Proteins were subsequently transferred onto a PVDF membrane for 2 h at 4°C and then blocked with tris-buffered saline–0.1% Tween 20 (TBST) containing 5% nonfat dry milk for 2 h at room temperature. After blocking, the membrane was incubated with primary antibody for 12 h at 4°C. NPM and GSTA3 antibodies were from Abcam (Cambridge, U.K.). The GNMT antibody was from Santa Cruz Biotechnology (California, U.S.A.). The PVDF membrane was washed five times (10 min each) with TBST and then incubated for 1 h with secondary antibody (Beyotime Biotechnology, ShangHai, China). Antibody binding was detected by enhanced chemiluminescence (ComWin Biotech Co., Ltd., Beijing, China). Chemical luminescence reagent was used to detect Western blotting (WB) results.

### Statistics

All statistical analyses were performed with the SPSS statistical software, version 18.0. And the results were presented as S.D. ± mean. We used *t*-test to test the differences between groups and *P*<0.05 was considered significant.

## Results

### Morphological changes in the liver tissues of mice that were exposed to different radiation dose rates originating from UT

**Figure 1:**
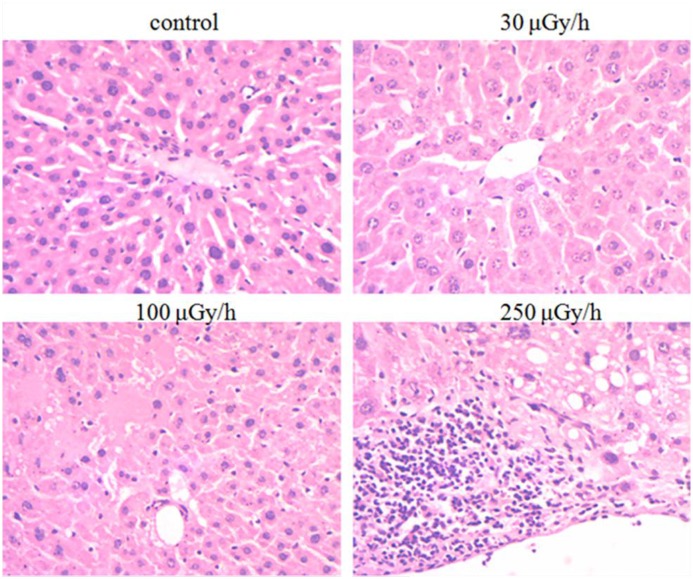
Morphology of liver tissues obtained from mice that were chronically exposed to 30, 100, and 250 μGy/h

Histological analysis of mouse liver tissue obtained from animals that received different dose rates of UT irradiation over a 500 day period was performed to determine whether any gross change in tissue morphology had occurred. As shown in Figure 1, the central vein was slightly dilated in the 30 μGy/h irradiated group, when compared with the matched control group. We did not observe other pathological changes in the liver tissues. Central vein dilation was obvious in the 100 and 250 μGy/h groups. Necrosis was also prominent in the liver tissues of the 250 μGy/h group, and there was also some interstitial fibrosis and steatosis.

### Comparative proteomics analysis of nonirradiated and irradiated mice by 2-DE

We detected more than 600 protein spots in each gel. There were 10, 11, and 17 spots above the 2.5-fold-change threshold in the 30, 100, and 250 μGy/h experimental groups, respectively, when compared with the matched control group.

### Mass spectrometry and bioinformatics analysis of the differentially expressed protein spots

**Figure 2:**
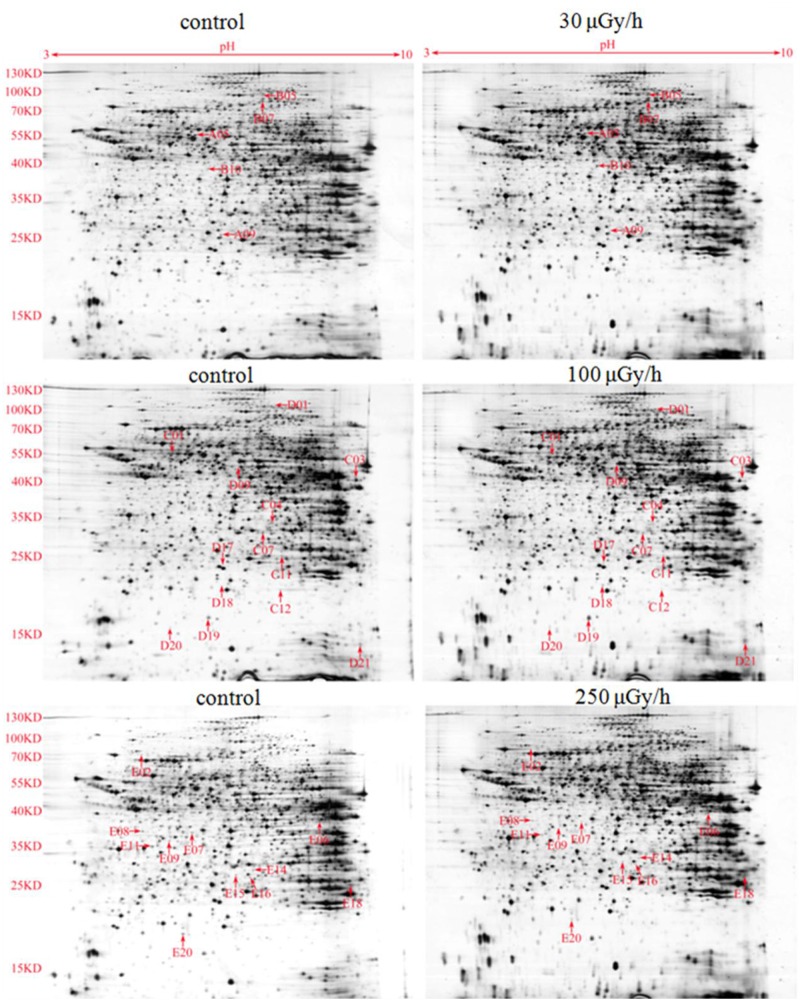
2-DE of total liver protein obtained from mice that were chronically exposed to 30, 100, and 250 μGy/h

**Figure 3:**
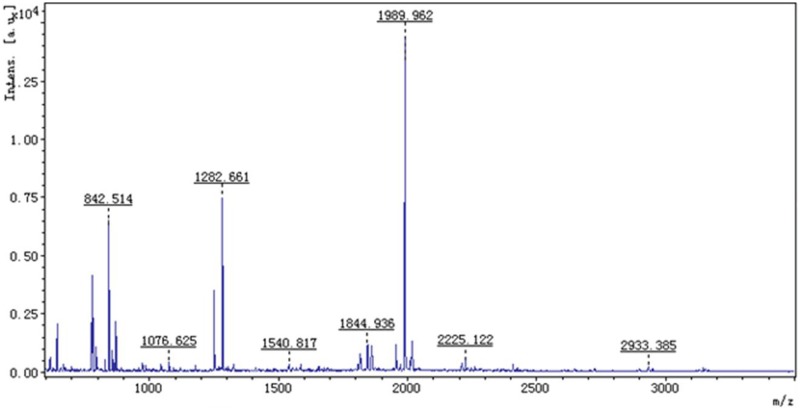
PMF print of protein spot C04 obtained using MALDI-TOF-MS

We used matrix-assisted laser desorption/ionization time of flight mass spectrometry (MALDI-TOF-MS) to analyze the 29 protein spots that satisfied our selection criteria (≥2.5-fold changes in expression with obvious error dots removed), and then performed a database search to identify individual proteins. Figure 2 shows 29 spots with accurate spectral information provided by the PD Quest Software. Figure 3 provides the peptide mass finger print of spot number C04 obtained by MALDI-TOF-MS. The SWISS-PROT and NCBI databases were used to analyze 24 spots. Protein spot number C04 was identified as GNMT, with a score of 236 and a sequence coverage of 45%. Eighteen protein spots had a score of more than 55, and their data are summarized in Table 2.

**Table 2:** Differentially expressed proteins identified by MALDI-TOF-MS

Spot	Entry name	Protein identified	Score	Mr (Da)	IP	Squence coverge	Function
09	IDI1_MOUSE	Isopentenyl-diphosphate Delta-isomerase 1*	166	26615	5.79	37%	Catalysis
B07	CPSM_MOUSE	Carbamoyl-phosphate synthase [ammonia], mitochondrial^†^	235	165711	6.48	8%	Catalysis
C01	K2C8_MOUSE	Keratin, type II cytoskeletal 8*	172	54531	5.7	22%	Cytoskeletal protein/protection
C04	GNMT_MOUSE	Glycine N-methyltransferase *	236	33111	7.1	45%	Metabolic enzyme regulates lipid and glucose homeostasis
C07	D3YU12_MOUSE	NmrA-like family domain-containing protein 1*	150	33232	6.32	25%	Domain protein
C11	CMBL_MOUSE	Carboxymethylenebutenolidase homolog*	140	28226	6.71	32%	Hydrolases
C12	QOR_MOUSE	Quinone oxidoreductase*	177	35531	8.18	18%	Chemical carcinogen metabolizing enzyme
D18	GSTP1_MOUSE	Glutathione S-transferase P 1^†^	265	23765	7.68	42%	Liver detoxification
D19	GSTA3_MOUSE	Glutathione S-transferase A3^†^	171	25401	8.76	55%	Carcinogenesis
D20	A0A087WQI6_MOUSE	Glutathione S-transferase (Fragment)^†^	60	15359	5.38	8%	Liver detoxification
D21	BHMT1_MOUSE	Betaine–homocysteine S-methyltransferase 1^†^	172	45448	8.01	20%	Zinc-dependent enzyme/catalysis
E08	NPM_MOUSE	Nucleophosmin*	72	32711	4.62	23%	Cell growth, proliferation, and apoptosis/carcinogenesis
E09	ALBU_MOUSE	Serum albumin*	198	70700	5.75	21%	Maintaining the oncotic pressure/free radical scavenging
E11	TBB5_MOUSE	Tubulin β-5 chain*	136	50095	4.78	14%	Cytoskeletal protein
E14	HUTH_MOUSE	Histidine ammonia-lyase*	207	72897	5.94	20%	Catalysis
E15	GNMT_MOUSE	Glycine N-methyltransferase*	230	33111	7.1	34%	Metabolic enzyme regulates lipid and glucose homeostasis
E16	HGD_MOUSE	Homogentisate 1,2-dioxygenase*	123	50726	6.86	17%	Catalysis
E20	Q9CPX4_MOUSE	Ferritin light chain 1*	378	20817	5.66	65%	Iron metabolism

*Up-regulated compared with the control group. ^†^Down-regulated compared with the matched group.

### Real-time polymerase chain reaction verification

We chose GNMT, GSTA3, and NPM for further analysis and performed real-time PCR to verify that changes observed at the protein level were reflected by corresponding changes in gene expression. Proteomics had revealed that GNMT was up-regulated in both the 100 and 250 μGy/h irradiated groups, that GSTA3 was down-regulated in the 100 μGy/h irradiated group, and that NPM was up-regulated in the 250 μGy/h irradiated group.

**Figure 4:**
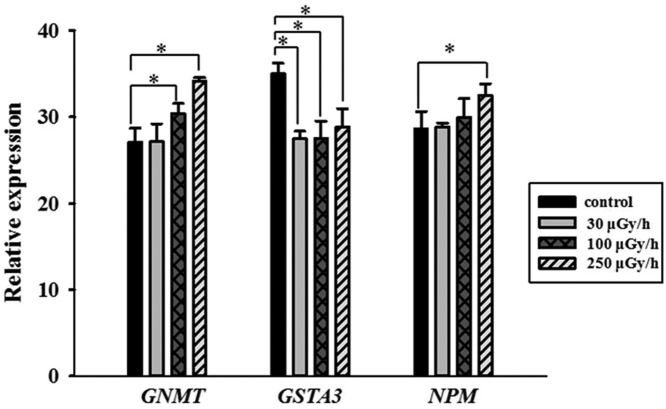
qPCR verification of GNMT, GSTA3, and NPM gene expression in liver obtained from mice that were chronically exposed to 30, 100, and 250 μGy/h.

As shown in Figure 4, GNMT mRNA expression was increased in both the 100 and 250 μGy/h irradiated groups, when compared with controls (*P*<0.05), a finding that was consistent with the proteomics analysis. GSTA3 mRNA expression, however, was decreased in all γ-irradiated groups (*P*<0.05). NPM mRNA expression was increased in the 250 μGy/h irradiated group, when compared with controls (*P*<0.05), again findings that were consistent with proteomics analysis.

Values are presented as the mean ± S.D. from three experiments. We used *t*test to test the differences between groups and *P< 0.05 was considered significant.

### WB verification

**Figure 5:**
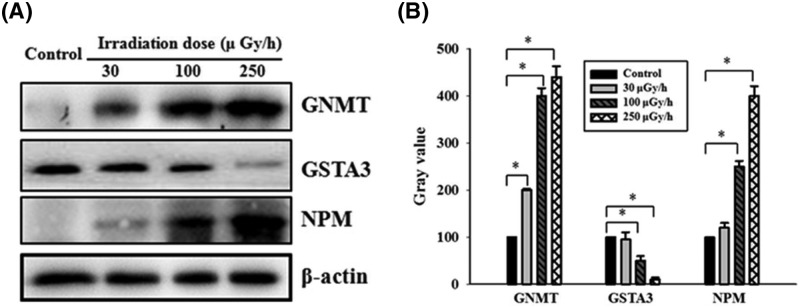
WB verification of GNMT, GSTA3, and NPM expression in liver obtained from mice that were chronically exposed to 30, 100, and 250 μGy/h. (**A**) Representative WB of GNMT, GSTA3, and NPM expression in the liver tissues of mice treated with or without the indicated doses of irradiation (β-actin served as the internal control). (**B**) Quantification of GNMT, GSTA3, and NPM expression, evaluated by WB analysis. Values are presented as the mean ± S.D. from three experiments. We used *t*test to test the differences between groups and *P<0.05 was considered significant.

We next performed WB studies to verify the γ-irradiation-dependent changes in the protein expression of GNMT, GSTA3, and NPM observed in our proteomics analysis. WB analysis revealed that, after 500 days of irradiation, GNMT and NPM protein expression was elevated and GSTA3 expression was decreased in liver tissues, when compared with controls (Figure 5). These changes in expression were consistent with the results of the proteomics analysis.

## Discussion

Humans are unavoidably exposed to low doses of radiation. Recently, increased attention has been given to biological effects of low dose rate or LDR on normal tissues. It is therefore desirable for radiation protection that low dose rate biomarkers be developed. The study of low dose and low dose rate provides guidance for radiation protection [25]. And the study of health effects of LDR is important for radiological protection research [26]. It follows that understanding the cellular responses to low dose rate is important. From previous studies it is known that it is difficult to detect an increase in DNA damage when the total cumulative dose is 400 times higher than the natural background and that doses are delivered at low dose rate. However, DNA damage can be easily measured when doses are delivered at high dose rate [27]. Another study showed that radiation genotoxicity depends on the dose rate [28]. Thus, dose rate is an important parameter to consider when developing radiation protection standards and for estimating risks.

Many researcher groups have provided fundamental data on the risk of ionizing radiation exposure for cancer development in humans [29]. The effects of LDR on an organism may include DNA damage and chromosome aberrations [30,31].

In our current study, we exposed mice to different doses of UT radiation for 500 days and then performed a proteomics analysis to identify possible changes in protein expression in the livers of the irradiated animals, given the observed effects of LDR on this organ. An analysis of differentially expressed proteins in cells and tissues under different conditions has previously proved useful in the identification of biomarkers associated with LDR [32,33].

We selected 29 protein spots based on the 2-DE gel image results for subsequent MALDI-TOF-MS analysis. Eighteen of these proteins were characterized successfully. Thirteen of the eighteen proteins related to low dose irradiation were up-regulated in the mouse liver and five were down-regulated. These data are summarized in Table 2. These proteins are related to detoxification, tumorigenesis, catalysis, cytoskeletal processes, and metabolism. Our research has shown that GNMT expression is enhanced in the liver tissues of mice following *in vivo* exposure to radiation originating from UT. GNMT is an important protein involved in folate metabolism, a process in which methionine and methylation play an important role [34]. In mammals, GNMT expression differs in various organs and tissues, suggesting that GNMT has tissue-specific roles. GNMT has been implicated in a variety of cancers. For example, it is down-regulated in almost all hepatocellular carcinoma (HCC) [35]; although abundantly expressed in the normal liver, GNMT expression is decreased in HCC tissues. GNMT deficiency is also associated with an inherited disorder of methionine metabolism [36]. Hepatic GNMT regulates lipid and glucose homeostasis, and its deficiency results in increased lipogenesis and triglycerides [37]. The overexpression of GNMT has been shown to prevent aflatoxin-induced carcinogenicity and to inhibit liver cancer cell proliferation. And the overexpression of GNMT provides insight into the development of insulin resistance through the modulation of the PI3K/Akt pathway [38]. The physiological role of GNMT can also provide insight into its connection with prostate cancer (PC) at various levels, including gene structure, gene expression, and metabolism. Moreover, GNMT plays an important role in controlling the methylation status of cells and in the metabolism of folic acid and methionine. During sarcosine treatment, GNMT expression in PC was observed to be affected slightly [39]. In our study we have shown that GNMT expression is altered following the 500-day irradiation with UT at all dose rates tested. GNMT can therefore be considered a promising target in the development of advanced diagnostic and/or prognostic techniques.

GSTA3 is a member of the glutathione S-transferase (GST) family, which is the most catalytically efficient steroid isomerase enzyme known in humans. GSTA3 plays an important role in the synthesis of steroid hormones [40–48], and it is selectively expressed in steroidogenic tissues. Mice GSTA3 mRNA levels were found to be elevated in animals after acute exposure to 8 Gy of γ-ray [49]. Given that this enzyme is a member of the GST family, this observation may reflect its radioprotective properties. In the present study, we found that GSTA3 expression was decreased in the three treatment groups exposed to different dose rates of UT irradiation. These results indicated that GSTA3 expression in the liver was altered in response to irradiation by UT.

NPM is mainly located in the nucleolus of cells and is an important protein implicated in many solid tumors. In the present study, NPM expression was found to be up-regulated in the experimental groups, when compared with the nonirradiated control group. In previous reports, NPM has been associated with tumor resistance and progression, and NPM silencing has been shown to significantly enhance the antitumor effects of baicalin, a natural component of the flowering plant, Scutellaria baicalensis Georgi [50]. NPM plays a role in promoting cancer, and its nucleolar localization is important for its cancer-promoting properties. Influencing the nucleolar-to-cytoplasmic distribution of this protein may therefore provide a potential strategy for HCC treatment [51]. NPM expression predicts recurrence and survival in upper tract urothelial carcinoma. High NPM expression significantly correlates with tumor location [52]. NPM is a multifunctional oligomeric phosphoprotein and is encoded by the *NPM1* gene [53,54]. NPM1mA is a mutant form of the NPM1 gene and is the most common genetic abnormality in patients with acute myeloid leukemia (AML) [55,56]. NPM1mA functions as an oncogene and is present in ∼30% of patients with AML [57]. Wild-type NPM1 is also frequently overexpressed in various tumors. NPM1 knockdown has been shown to significantly decrease nuclear factor-κB-mediated invasion of breast cancer cells. Nuclear factor-κB is a master regulator of inflammation [58]. Targeting NPM may provide a novel and effective method of reversing the effects of multidrug resistance, and this protein may be a potential adjuvant for tumor chemotherapy [59].

However, the biological effects of LDR are still unclear. The hazards associated with UT are numerous given its possible contribution to water pollution and acid rain, in addition to the internal risk that it can pose to the human body. Our study reveals that GNMT, GSTA3, and NPM are involved in the stress response induced in the liver tissues of mice following a 500-day exposure to less than or equal to 250 μGy/h originating from UT.

## Abbreviations

2-DE: two-dimensional gel electrophoresis
AML: acute myeloid leukemia
BCA: bicinchoninic acid
GNMT: glycine N-methyltransferase
GST: glutathione S-transferase
GSTA3: glutathione S-transferase A3
HCC: hepatocellular carcinoma
IPI: international protein index
LDR: low dose radiation
MALDI-TOF-MS: matrix-assisted laser desorption/ionization time of flight mass spectrometry
NPM: nucleophosmin
PC: prostate cancer
PCR: polymerase chain reaction
PI3K/Akt: phosphatidylinositol-3 kinase/Akt
PMF: peptide mass fingerprinting
PVDF: polyvinylidene fluoride
S.D.: standard deviation
RT: real-time
TBST: tris-buffered saline–0.1% Tween 20
UT: uranium tailings
WB: Western blotting
